# Effects of Different Sources of Calcium in the Diet on Growth Performance, Blood Metabolic Parameters, and Intestinal Bacterial Community and Function of Weaned Piglets

**DOI:** 10.3389/fnut.2022.885497

**Published:** 2022-04-29

**Authors:** Anqi Yang, Kaijun Wang, Xiaomin Peng, Feifei Lv, Ying Wang, Yao Cui, Yuhan Wang, Dongshuai Qu, Jianqun Zhou, Hongbin Si

**Affiliations:** ^1^State Key Laboratory for Conservation and Utilization of Subtropical Agro-Bioresources, College of Animal Science and Technology, Guangxi University, Nanning, China; ^2^Animal Nutritional Genome and Germplasm Innovation Research Center, College of Animal Science and Technology, Hunan Agricultural University, Changsha, China; ^3^Nanning Zeweier Feed Co., Ltd., Nanning, China

**Keywords:** calcium, pig, bacterial community, intestine, metabolites

## Abstract

Despite a well-documented effect of calcium on the piglet's intestinal microbiota composition, it is less known about changes in microbial function or the effect of different sources of calcium. The experiment was designed to study the effects of dietary calcium from different sources on production, immune indexes, antioxidant capacity, serum biochemical indexes, and intestinal microflora of weaning piglets. A total of 1,000 piglets were randomly assigned to five groups (10 replicate pens per treatment with 20 pigs per pen) and fed diets supplemented with calcium carbonate, calcium citrate, multiple calcium, organic trace minerals, and different concentrations of acidifier. The results showed that the replacement of calcium carbonate with calcium citrate and multiple calcium had almost no significant difference in the growth performance of pigs compared with the control group, and only the diet of multiple calcium dramatically decreased the average daily feed intake (ADFI) compared to the calcium citrate diet on days 15–28 (*p* < 0.05). The five groups did not change the content of MDA, SOD, and GSH-Px (*p* > 0.10). A similar situation occurs in the immune function of the blood. There was no significant effect in immune indexes (IgA, IgG, and IgM) among different treatments after weaning at 6 weeks for piglets (*p* > 0.10). The 16S rRNA sequencing of ileal and cecal microbiota revealed that only the relative abundance of *Actinobacteriota* at the phyla level was significantly greater in the ileum of the A group compared to the other treatments (*p* < 0.05). There was a clear effect on seven bacteria in the top 30 genera of ileum and cecum for five groups (*p* < 0.05). The result of PICRUSt predicted that the intestinal microbe was mainly involved in carbohydrate and amino acid metabolism, membrane transport, and metabolism of cofactors and vitamins. Besides, adding calcium citrate to a weaned piglet diet is better than other choices from the third week to the fourth week. In conclusion, diets with different calcium sources changed ADFI and some intestinal microbial composition of weaned piglets but had little effect on intestinal microbial function.

## Graphical Abstract

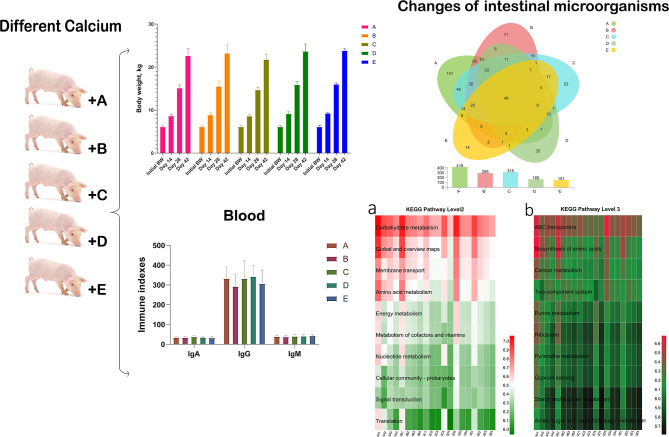

## Introduction

The weaning transition period of piglets is related to reduced feed intake, poor production performance, and postweaning diarrhea (1, 2). The mammalian gastrointestinal tract contains a complex microbiota that provides the animal with several protective and metabolic functions, including the development and regulation of the immune system, the absorption of nutrients from some indigestible polysaccharides, and the competitive exclusion of pathogens (3–5). Minerals and vitamins are trace substances essential to the normal physiological function of animals, but their effects on intestinal flora are not well-understood. A study has proved that high zinc has a significant effect on anti-diarrhea in piglets, which is closely related to the significant decrease in the amount of *Escherichia coli* in feces, and Zinc ions can reduce the harmful effects of *E. coli* by inhibiting its respiratory chain (6). However, some studies have suggested that adding high zinc to piglets can increase the number of *E. coli* and *Enterococcus*, while adding high copper can inhibit the growth of intestinal harmful bacteria in piglets (7). Based on studies in rats, dietary supplementation of calcium and phosphorus has been suggested as a potential strategy to modulate the gastrointestinal tract microbiota in pigs. Studies have shown that diets rich in calcium and phosphorus reduce the number of pathogens and increase the number of lactobacilli in the gut (8, 9).

Gut microbes play a key role in animal health, including digesting food, metabolism, regulating immunity, and defending against invading pathogens (10–15). There are three main types of microorganisms in the digestive tracts of monogastric animals, which are bacteria, archaea, and eukaryotes. Bacteria are dominant in number and are mainly anaerobic bacteria (16, 17). The microorganisms in the intestines of pigs are mainly anaerobic bacteria and facultative anaerobic bacteria, of which Firmicutes and Bacteroidetes account for more than 90% and play an important role in maintaining body health and improving body immunity, nutrient absorption, and metabolism (18). The composition of different microbial communities in the digestive tracts of animals is different, and the diversity and density of microbial communities increase gradually from the stomach to the hindgut (19). The cecum is the place with the most abundant microorganism species and content in single-stomach animals. The number of microbes per gram of intestinal content in pigs is 10^12^-10^13^ CFU and is composed of 400–500 species of microorganisms, which mainly consist of *Bacteroides* (8–28%) and *Clostridium* X and IV of Firmicutes (10–29%), with *Clostridium* IV (25.2%) as the predominant flora (20–22). Stable intestinal microbial flora can form a bacterial membrane barrier on the surface of intestinal epithelial cells to help the host resist harmful foreign bacteria and inhibit the invasion and reproduction of intestinal pathogenic bacteria by competing for nutrients (23). At the same time, the stable microbial flora in the intestine participates in the host's nutrient metabolism through fermentation, degradation of polysaccharides, and synthesis of vitamins (24, 25). The main purpose of this study was to assess the impact of different combinations of post-weaning calcium and acidifier supplementation on the production performance, blood metabolism, and microbiota structure and function of the ileal and cecal contents of weaned piglets.

## Experimental Design

All experimental procedures involving animals were approved by the Laboratory Animal Welfare and Animal Experimental Ethical Inspection Committee at the Guangxi University (Nanning, China).

### Animals, Diets, and Management

A total of 1,000 piglets (Yorkshire × Landrace) were weaned at the age of 21 days with an average body weight (BW) of 6.09 ± 0.26 kg; they were assigned to 1 of 5 treatments with 20 replicate pens (50 piglets per replicate pen) for 42 days. Water and feed were provided *ad libitum*. The body weight and feed intake of pigs were recorded weekly.

The compositions of the basal diets are shown in Supplementary Table 1. An experiment diet (Table 1) was formulated to provide varying dietary calcium concentrations from calcium carbonate, calcium citrate, or multiple calcium. The formula of the diet should meet or exceed the nutritional needs of weaned piglets (26). B = A (control) plus 5/1,000 calcium citrate to replace calcium carbonate; C = B plus 1/1,000 organic trace minerals; D = C minus half of the acidifier; E = A (control) plus 5/1,000 multiple calcium to replace calcium carbonate. All piglets were fed five different diets for 6 weeks before being euthanized and samples collected. Piglets were housed on a 12-h light-dark cycle with free access to water, and the barn temperature was maintained at 30°C.

**Table 1:** Experimental diets for 1,000 weaned piglets.

**Items**	**Groups**				
	**A (***n*** = 200)**	**B (***n*** = 200)**	**C (***n*** = 200)**	**D (***n*** = 200)**	**E (***n*** = 200)**
Organic trace minerals (kg)	–	–	1/1,000	1/1,000	1/1,000
Acidifier (kg)	1/1,000	1/1,000	1/1,000	0.5/1,000	1/1,000
Calcium carbonate (kg)	Normal	–	–	–	–
Calcium citrate (kg)	–	5/1,000	5/1,000	5/1,000	–
Multiple calcium (kg)	–	–	–	–	5/1,000

*B = A (control) plus 5/1,000 calcium citrate replace calcium carbonate; C = B plus 1/1,000 organic trace minerals; D = C minus half of the acidifier; E = C plus 5/1,000 multiple calcium replace calcium carbonate. Organic trace minerals: as shown in Table 2; calcium carbonate: Ca ≥ 36.65%; calcium citrate: Ca ≥ 23.4%; multiple calcium: Ca ≥ 23.4%. The same as below*.

**Table 2:** Composition of organic trace minerals.

**Items**	**Content (g/kg)**
Calcium (Ca)	0
Ferrous fumarate (Fe)	150–200
Zinc fumarate (Zn)	90–140
Copper methionine (Cu)	12–15
Manganese methionine (Mn)	12–20
Potassium iodide (I)	0.2–0.8
Sodium selenite (Se)	0.2–0.45

### Sampling and Collection

All piglets and feeds were weighed on days 0, 14, 28, and 42 to calculate the average daily feed intake (ADFI), average daily gain (ADG), and feed to meat ratio (F:G). On day 42, two blood samples were collected using heparin tubes from the front cavity veins of eight weaned piglets in five groups separately. Collected plasma samples were centrifuged at 1,000 × *g* for 15 min at 4°C and stored at −20°C for further analysis. Four weaned piglets were sacrificed in each group, and intestinal samples were subsequently collected.

### Metabolites Measure in the Plasma

Two piglets were selected in each repeat of five groups (40 piglets in all), and a total of 40 plasma samples were used for analysis. The plasma biochemical components include malondialdehyde (MDA), glutathione peroxidase (GSH-Px), superoxide dismutase (SOD), immunoglobulin-A (IgA), immunoglobulin-G (IgG), immunoglobulin-M (IgM), alanine aminotransferase (ALT), aspartate aminotransferase (AST), amylase (AMY), alkaline phosphatase (ALP), and lactate dehydrogenase (LDH) were measured using commercially available kits (Jiangsu Meimian Industrial Co., Ltd, Yancheng, China) following the manufacturer's instructions.

### Ileum and Cecum Content Microflora 16S RRNA Sequencing

One piglet was selected randomly to execute (20 piglets in all) in each repeat of five groups and a total of 20 ileal samples and 20 samples of cecum for microbiota analysis. Microbial DNA was extracted from approximately 0.25 g of each intestinal chyme using a QIAamp DNA Stool Mini Kit (Qiagen, Germany), according to the manufacturer's instructions (27). Successful DNA isolation was performed by 2% of agarose gel electrophoresis. The bacterial universal V3–V4 region of the 16S rRNA gene was amplified according to PCR-barcoded primers 515F (5′-ACTCCTACGGGAGGCAGCAG-3′) and the reverse primer 806R (5′-GGACTACHVGGGTWTCTAAT-3′). The specific sequencing method was done as previously reported (28). The thermal cycle procedure is as follows: initial denaturation step, 95°C, 3 min; denaturation, 27 cycles, 95°C, 30 s; annealing, 55°C, 30 s; elongation, 72°C, 45 s; and final extension, 72°C, 10 min. Briefly, paired-end was sequenced on an Illumina MiSeq platform (PE300) platform (Illumina, USA) at the Majorbio Bio-Pharm Technology (Shanghai, China). The 16S rRNA amplicon sequences have been deposited in the National Center for Biotechnology Information (NCBI) Sequence Read Archive (SRA) (http://www.ncbi.nlm.nih.gov/bioproject/815951) under accession number PRJNA815951.

### Microbiome Analysis

Quality filters were applied to trim the original sequences according to the criteria: (I) reads with an average quality score <20 over a 10-bp sliding window were re-moved, and truncated reads smaller than 150 bp were discarded. (II) Truncated reads containing homopolymers longer than eight nucleotides in length, more than 0 bases in barcode matches, or more than two different bases in primers were removed from the dataset. Checking and removing possible chimeras by USEARCH using the chimera layer “gold” database described by Edgar et al. (29). Clustering of OTUs with a similarity cutoff of 97% using USEARCH (30) and abundance-defining representative sequences for each OTU were identified using PyNAST (31) and the SILVA bacterial database (32). The rarefaction analysis was performed using Mothur version 1.39.5 (33) to reveal diversity indices, including Chao index and Shannon index. PCoA was performed using Canoco 4.5. Venn diagrams were implemented by Venn Diagram, and community diagrams were generated using R tools from the data in the files “tax. phylum.xls, tax.family.xls, and tax.genus.xls.”

### Predictive Functional Profiling of Microbial Communities

PICRUSt has been used as a bioinformatics tool to predict the functional potential of metagenomes using 16S rRNA genetic data (34). Subsequently, by referencing the Kyoto Encyclopedia of Genes and Genomes (KEGG) database, the OTU table was imported into PICRUSt for functional gene prediction. PICRUSt utilizes 16S copy number prediction to normalize the OTU table so that OTU abundance more accurately reflects the true abundance of the underlying organism. We then looked for the precomputed genome content of each OTU, multiplied the normalized OTU abundance by each KEGG abundance in the genome, and summed these KEGG abundances for each sample to predict the metagenome. This prediction calculates the KEGG abundance for each metagenomic sample in the OTU table. For those optional organism-specific predictions, each organism's abundance per KEGG is kept and annotated. We focused our exploration of metagenomes on levels 2 and 3. These pathways related to organismal systems, human disease, and drug development are filtered out because they do not reflect microbial function.

### Statistical Analyses

Statistical analyses between the means of each group were analyzed using one-way ANOVA (one-way analysis of variance) followed by multiple comparisons using a *post hoc* test of S-N-K through SPSS 22.0. Statistical significance was set at *p* < 0.05.

## Results

### Production Performance

Initial BW of weaned piglets did not differ among the 5 groups (Table 3). There were no significant effects of including different sources of calcium in five diets with normal or halved acidifier-fed piglets on BW in 6 weeks (*p* > 0.05). But the diet of multiple calcium dramatically decreased the ADFI compared to the C and D diet on days 15–28 (*p* < 0.05). Meanwhile, no significant differences in ADFI were noted between groups on days 1–14 and 29–42 (*p* > 0.10). As shown in Table 4, ADG increased significantly when multiple calcium instead of calcium citrate was added on days 1–14 (0.18 ± 0.03 vs. 0.22 ± 0.04). And F:G was not affected (*p* > 0.10) by the different sources of calcium in the diets at different time points for weaned piglets.

**Table 3:** Effects of different calcium sources on body weight and average daily feed intake of weaned piglets.

**Items**	**Times**	**Groups**				
		**A (***n*** = 200)**	**B (***n*** = 200)**	**C (***n*** = 200)**	**D (***n*** = 200)**	**E (***n*** = 200)**
BW, kg	Initial BW	6.09 ± 0.23	6.09 ± 0.25	6.09 ± 0.29	6.09 ± 0.31	6.09 ± 0.35
	Day 14	8.62 ± 0.43	8.86 ± 0.47	8.59 ± 0.31	9.11 ± 0.49	9.23 ± 0.29
	Day 28	15.1 ± 0.71	15.5 ± 1.30	14.7 ± 0.63	15.9 ± 0.77	16.0 ± 0.31
	Day 42	22.6 ± 1.67	23.2 ± 1.98	21.7 ± 1.13	23.7 ± 1.69	23.8 ± 0.47
ADFI, kg	Day 1–14	0.22 ± 0.02	0.24 ± 0.02	0.21 ± 0.04	0.24 ± 0.03	0.26 ± 0.03
	Day 15–28	0.67 ± 0.04^ab^	0.69 ± 0.07^ab^	0.71 ± 0.02^a^	0.72 ± 0.06^a^	0.63 ± 0.04^b^
	Day 29–42	0.94 ± 0.10	0.97 ± 0.09	0.89 ± 0.04	1.00 ± 0.11	0.96 ± 0.04

*B = A (control) plus 5/1,000 calcium citrate replace calcium carbonate; C = B plus 1/1,000 organic trace minerals; D = C minus half of the acidifier; E = C plus 5/1,000 multiple calcium replace calcium carbonate. BW, body weight; ADFI, average daily feed intake. In the same row, values with no letter or the same letter superscripts mean no significant difference (p > 0.05), while with different small letter superscripts mean significant difference (p < 0.05)*.

**Table 4:** Effects of different calcium sources on average daily gain and feed to meat ratio of weaned piglets.

**Items**	**Times**	**Groups**				
		**A (***n*** = 200)**	**B (***n*** = 200)**	**C (***n*** = 200)**	**D (***n*** = 200)**	**E (***n*** = 200)**
ADG, kg	Day 1–14	0.18 ± 0.02^ab^	0.20 ± 0.03^ab^	0.18 ± 0.03^b^	0.22 ± 0.02^ab^	0.22 ± 0.04^a^
	Day 15–28	0.46 ± 0.03	0.47 ± 0.06	0.44 ± 0.02	0.48 ± 0.03	0.48 ± 0.01
	Day 29–42	0.54 ± 0.07	0.55 ± 0.05	0.50 ± 0.04	0.56 ± 0.07	0.56 ± 0.03
F: G	Day 1–14	1.20 ± 0.09	1.21 ± 0.11	1.18 ± 0.04	1.13 ± 0.04	1.15 ± 0.09
	Day 15–28	1.44 ± 0.05	1.46 ± 0.06	1.45 ± 0.04	1.49 ± 0.07	1.46 ± 0.02
	Day 29–42	1.76 ± 0.07	1.75 ± 0.04	1.77 ± 0.06	1.80 ± 0.03	1.71 ± 0.05

*B = A (control) plus 5/1,000 calcium citrate replace calcium carbonate; C = B plus 1/1,000 organic trace minerals; D = C minus half of the acidifier; E = C plus 5/1,000 multiple calcium replace calcium carbonate. ADG, average daily gain; F:G, feed to meat ratio. In the same row, values with no letter or the same letter superscripts mean no significant difference (p > 0.05), while with different small letter superscripts mean significant difference (p < 0.05)*.

### Biochemical Parameters in the Plasma

Variation of the antioxidant index in the plasma is shown in Table 5. It was obvious that whatever piglets were fed, the calcium carbonate, calcium citrate, multiple calcium, or different amount of acidifier, none of them changed the content of MDA, SOD, and GSH-Px (*p* > 0.10). A similar situation occurs in the immune function of blood, and it has no significant difference in the immune index (IgA, IgM, and IgG) among different treatments after weaning 6 weeks for piglets (*p* > 0.10).

**Table 5:** Effects of different calcium sources on plasma antioxidant and immune indexes of weaned piglets.

**Items**	**Groups**				
	**A (***n*** = 8)**	**B (***n*** = 8)**	**C (***n*** = 8)**	**D (***n*** = 8)**	**E (***n*** = 8)**
MDA (nmol/L)	8.69 ± 1.33	8.90 ± 1.03	9.19 ± 1.01	9.08 ± 1.14	8.75 ± 1.13
SOD (U/L)	1,119 ± 253	1,120 ± 276	1,254 ± 361	1,085 ± 270	1,208 ± 288
GSH-Px (U/L)	165 ± 29	155 ± 23	151 ± 21	143 ± 25	161 ± 25
IgG (μg/ml)	333 ± 60	293 ± 63	333 ± 90	343 ± 56	306 ± 67
IgA (μg/ml)	34.94 ± 4.94	34.21 ± 4.59	38.04 ± 5.59	35.02 ± 4.97	32.41 ± 6.29
IgM (μg/ml)	39.46 ± 6.16	37.76 ± 6.05	40.73 ± 7.64	41.42 ± 7.18	42.88 ± 6.18

*B = A (control) plus 5/1,000 calcium citrate replace calcium carbonate; C = B plus 1/1,000 organic trace minerals; D = C minus half of the acidifier; E = C plus 5/1,000 multiple calcium replace calcium carbonate. MDA, malondialdehyde; SOD, superoxide dismutase; GSH-Px, glutathione peroxidase; IgG, immunoglobulin G; IgA, immunoglobulin A; IgM, immunoglobulin M. In the same row, values with no letter or the same letter superscripts mean no significant difference (p > 0.05), while with different small letter superscripts mean significant difference (p < 0.05)*.

Data of enzymes in the plasma of weaned piglets by feeding different calcium sources are displayed in Table 6. Comparing to the normal diet, the ALT concentration decreased significantly by feeding calcium citrate (B) in the diet for weaned piglets (165 ± 13 vs. 141 ± 23), but by adding additional organic trace minerals (C), the ALT concentration returned to the normal level (165 ± 13 vs. 164 ± 21). However, reducing the amount of acidifier in the diet by half (D) or replacing it with multiple calcium (E) does not change AST concentration (*p* > 0.05). Meanwhile, there was no statistically significant change in AST (*p* = 0.935), AMY (*p* = 0.242), and LDH (*p* = 0.524) concentrations in plasma among the five-diet fed piglets. Regarding ALP concentration in the plasma, no significant difference was observed when calcium citrate was in the diet instead of calcium carbonate (*p* > 0.05). In comparing diets based on calcium citrate, after half of the acidifier in the experimental diet for piglets, the concentration of ALP decreased dramatically (151 ± 24 vs. 125 ± 20) in the blood of weaned piglets. But when multiple calcium replaces the same amount of calcium citrate, there is no statistically significant change in the concentration of ALP (*p* > 0.05).

**Table 6:** Effects of different calcium sources on enzymes in plasma of weaned piglets.

**Items**	**Groups**				
	**A (***n*** = 8)**	**B (***n*** = 8)**	**C (***n*** = 8)**	**D (***n*** = 8)**	**E (***n*** = 8)**
ALT (U/L)	165 ± 13^a^	141 ± 23^b^	164 ± 21^a^	150 ± 20^ab^	158 ± 13^ab^
AST (U/L)	37.4 ± 5.9	37.3 ± 6.8	39.5 ± 6.4	37.2 ± 5.8	38.2 ± 5.2
AMY (U/L)	116 ± 18	122 ± 15	117 ± 17	123 ± 17	105 ± 19
ALP (U/L)	136 ± 25^ab^	144 ± 27^ab^	151 ± 24^a^	125 ± 20^b^	156 ± 19^a^
LDH (U/L)	89.8 ± 15	96.5 ± 20	101 ± 17	86.3 ± 14	92.0 ± 22

*B = A (control) plus 5/1,000 calcium citrate replace calcium carbonate; C = B plus 1/1,000 organic trace minerals; D = C minus half of the acidifier; E = C plus 5/1,000 multiple calcium replace calcium carbonate. ALT, alanine aminotransferase; AST, aspartate aminotransferase; AMY, amylase; ALP, alkaline phosphatase; LDH, lactate dehydrogenase. In the same row, values with no letter or the same letter superscripts mean no significant difference (p > 0.05), while with different small letter superscripts mean significant difference (p < 0.05)*.

### Ileal and Cecal Bacterial Diversity and Similarity

**Figure 1:**
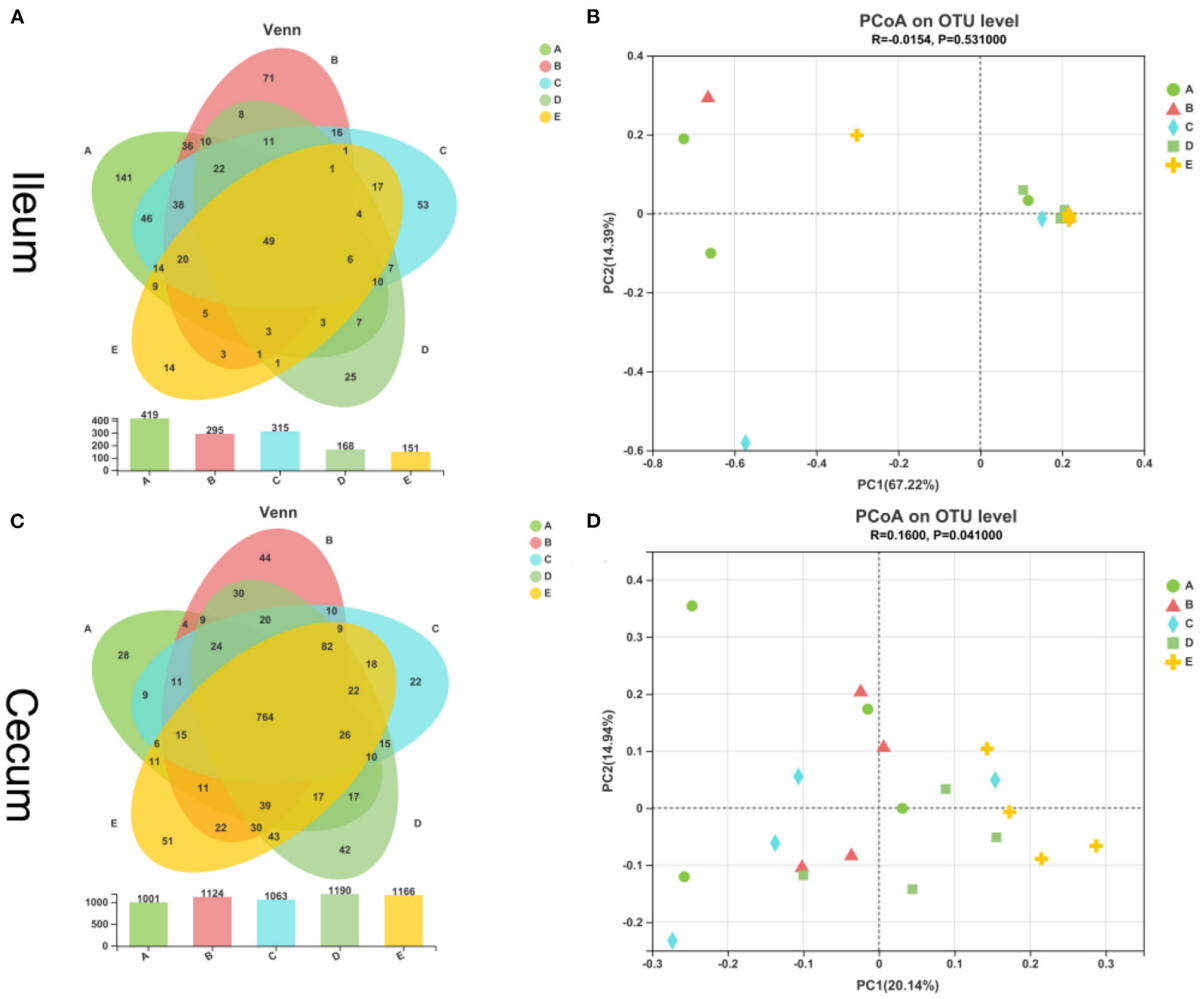
Similarity of intestinal bacterial community of weaned piglets. **(A)** Venn of the OTUs in the ileum by different treatments. **(B)** Principal coordinates analysis (PCoA) of ileal digestal bacterial community. **(C)** Venn of the OTUs in the cecum by different treatments. **(D)** Principal coordinates analysis (PCoA) of cecal digestal bacterial community.

As shown in Figure 1A, the overall OTU numbers classified on the distance level of 0.03 were 652 detected in the ileal samples, most abundance 419 OTUs in the control group observed and the E group has the least 151 OTUs, and 49 were shared among five groups. The cecal OTUs numbers were more than ileum and owned 1,461 OTUs (Figure 1C). On the contrary, the cecum control group had fewer OTUs than the ileum control group. And 764 OTUs were shared in the cecum by five diet treatments. Besides, the PCoA showed that 20 samples from the ileum in five groups were not separated completely (Figure 1B). In the cecal samples, we could see the E group stay away from the other four groups, and the other four sets of samples were mixed together (Figure 1D).

**Figure 2:**
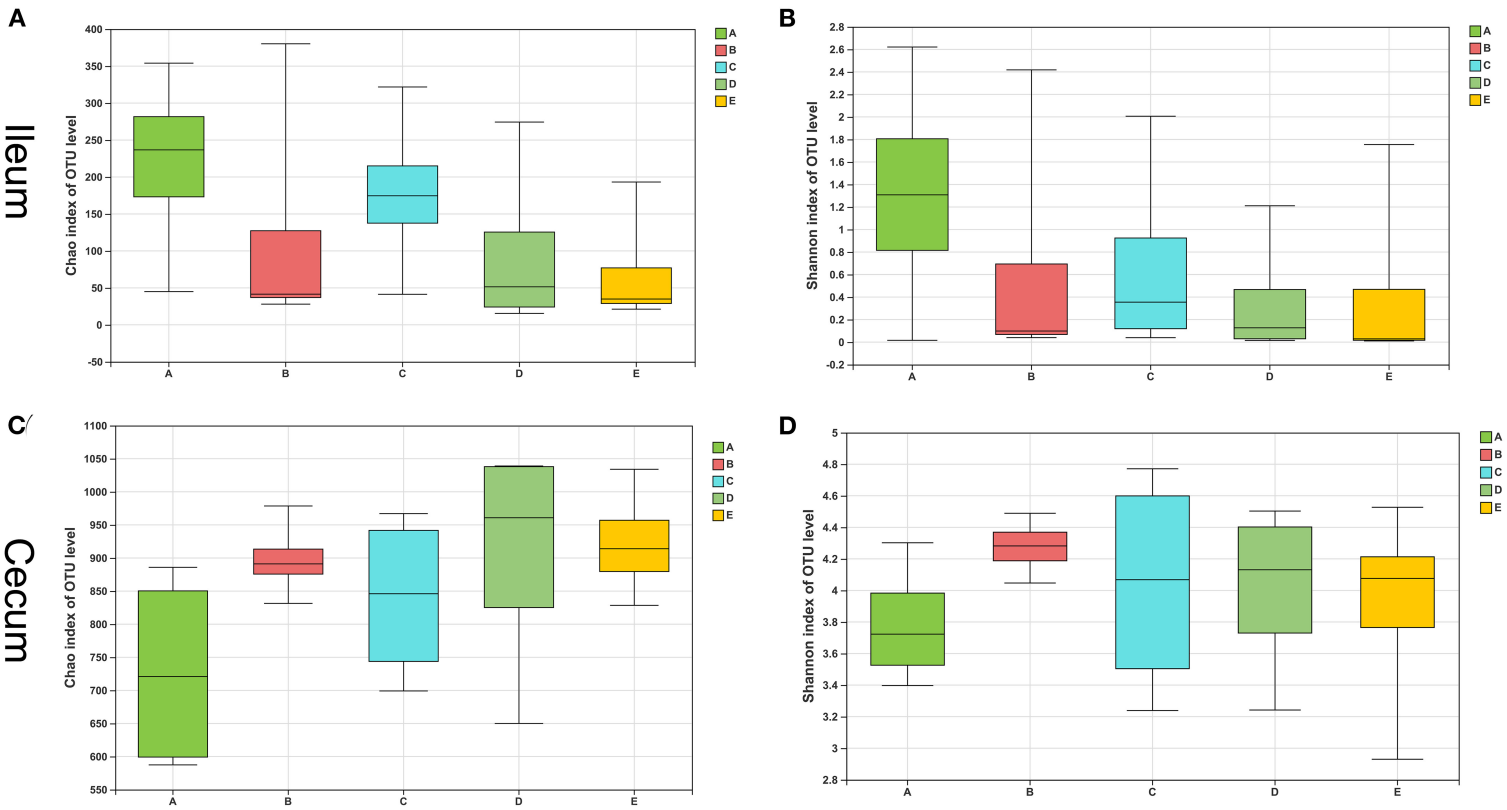
Alpha-diversity of intestinal bacterial community of weaned piglets. **(A)** The bacterial richness in the ileum estimated by the Chao1 value. **(B)** The bacterial diversity in the ileum estimated by Shannon index. **(C)** The bacterial richness in the cecum estimated by the Chao1 value. **(D)** The bacterial diversity in the cecum estimated by Shannon index.

The bacterial composition of the control group had a higher Chao1 estimator and Shannon diversity index than the other four groups, and the E group showed the lowest in the ileum (Figures 2A,B). However, the difference between the two indicators in the five groups was not statistically significant (*p* > 0.05). In contrast, the control group showed the lowest Chao1 estimator and Shannon diversity index in the cecum than the other four diet treatment groups (Figures 2C,D), and there was also no dramatic difference in the two indicators between the five groups (*p* > 0.05).

### Ileal and Cecal Bacterial Community Structure

**Figure 3:**
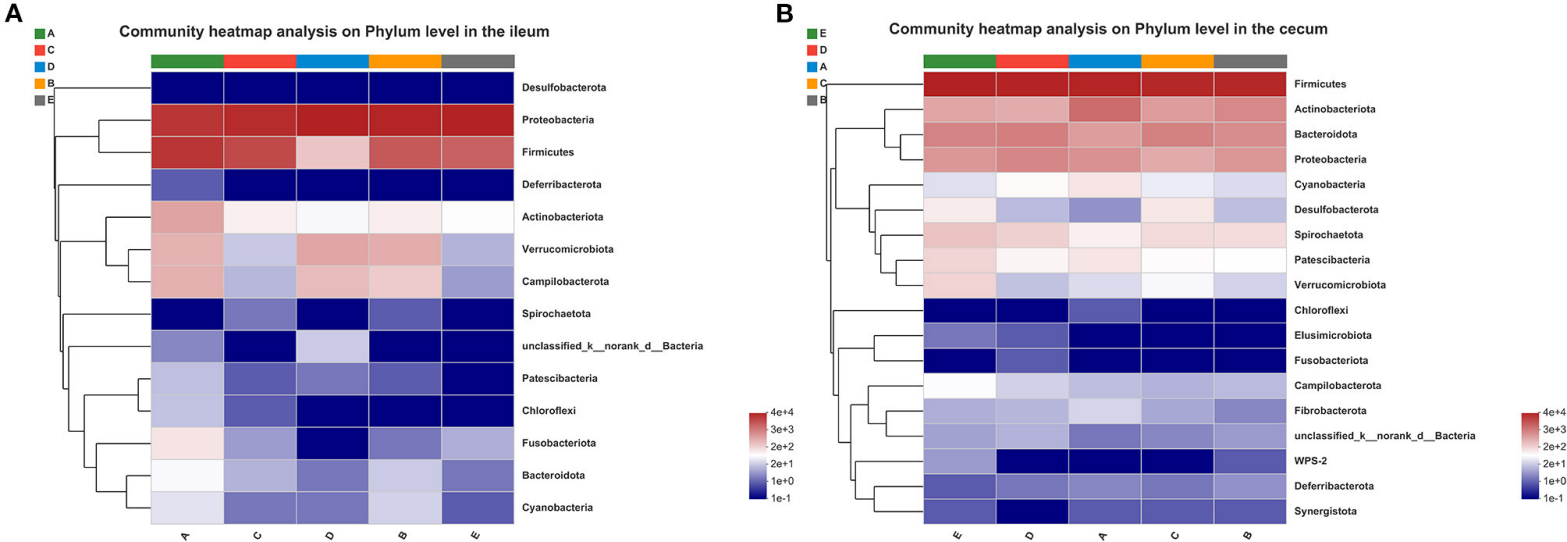
Effects of different diets on intestinal bacterial community structure in weaned piglets. **(A)** Distribution of ileal bacteria at phylum level in weaned piglets. **(B)** Distribution of cecal bacteria at phylum level in weaned piglets.

**Figure 4:**
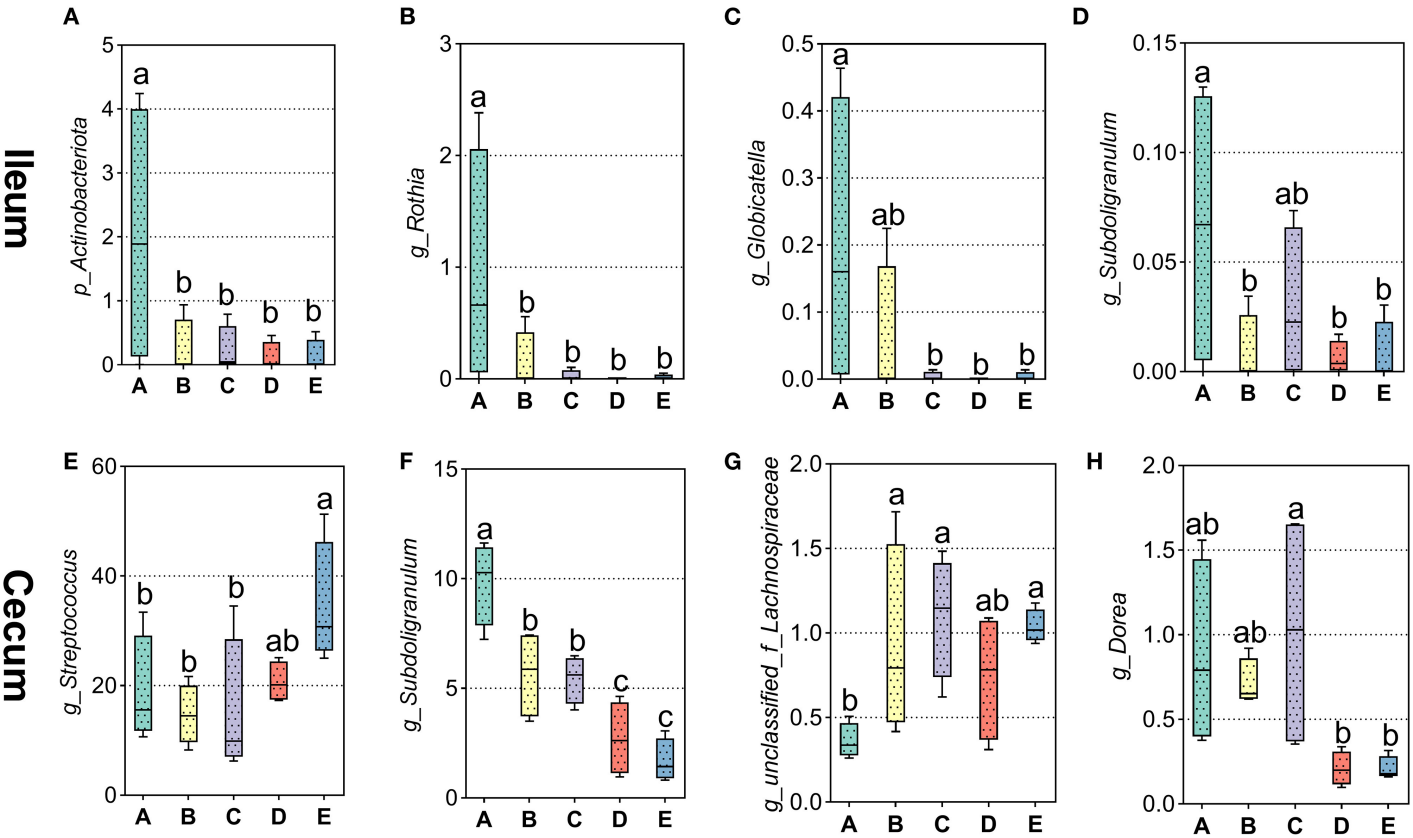
Effects of different diets on different intestinal bacteria in weaned piglets. **(A–D)** Distribution of ileal different bacteria at phylum level and top 30 different bacteria at genus level in weaned piglets. **(E–H)** Distribution of cecal top 30 different bacteria at genus level in weaned piglets.

As shown in Figure 3A, Proteobacteria, Firmicutes, Verrucomicrobia, Campilobacterota, and Actinobacteriota were dominant phyla in the ileum of weaned piglets, accounting for more than 90% of the total number of ileal bacteria. As shown in Figure 4A, only the abundance of Actinobacteriota at the phyla level was significantly greater in the ileum of the A group compared to the other groups (*p* < 0.05). Compared with in the ileum, the most abundant bacterial community at the phylum level was Firmicutes, followed by, from most to least, Bacteroidota, Actinobacteriota, Proteobacteria, and Spirochaetota (Figure 3B). Although the phylum proportions of some bacterial communities vary according to the different sources of calcium added to the diet, there was no statistical difference between the main bacterial communities at the phyla level in the cecum (*p* > 0.05).

Downward to genus levels, *Escherichia-Shigella, Streptococcus, Lactobacillus, Klebsiella*, and *Actinobacillus* were the dominant genus in the ileum in the five groups (Supplementary Figure 1). In Figure 4B, the relative abundance of *Rothia* was significantly higher in the ileum of the A group compared to the other groups (*p* < 0.05). Analogously, the *Globicatella* (Figure 4C) and *Subdoligranulum* (Figure 4D) were significantly greater in the ileum of the A group compared to the D and E groups, respectively (*p* < 0.05). In the cecum, the top five genera in bacterial abundance were *Streptococcus, Lactobacillus, Clostridium_sensu_stricto*_1, *Subdoligranulum*, and *UCG*-005 (Supplementary Figure 2). In Figure 4E, the relative abundance of *Streptococcus* was significantly greater in the cecum of the E group in weaned piglets by multiple calcium diet than in the A, B, and C groups (*p* < 0.05). On the contrary, the abundance of Subdoligranulum (Figure 4F) decreased significantly when weaned piglets were fed the D and E diets than A, B, and C groups (*p* < 0.05). Besides, the abundance of *unclassified_f_Lachnospiraceae* (Figure 4G) was lower in the cecum of the A group than in the other groups (*p* < 0.05). And the abundance of Dorea (Figure 4H) decreased significantly when weaned piglets were fed the D and E diets than A, B, and C groups (*p* < 0.05).

### Function Prediction of Ileal and Cecal Microbiota Using PICRUSt

In the study, PICRUSt was used to analyze the microbiota function of the ileum and cecum. The 16S rRNA sequencing results combined with genomic databases could be used to predict macrogenomic information (34). The predictable outcomes can be enriched at 2 and 3 levels of the KEGG pathways in the ileum (Supplementary Figure 3). In the top 10 KEGG pathways shown in Supplementary Figure 3a, membrane transport and signal transduction pathways were associated with environmental information processing. Six other pathways, including the metabolism of carbohydrates, global and overview maps, amino acids, energy, nucleotides, cofactors, and vitamins were associated with nutrients metabolism. The prokaryotic cellular community was associated with cellular processes. Totally, 320 pathways were predicted, and the top 10 pathways consisted of two pathways related to carbohydrate metabolism (as shown in Supplementary Figure 3b), including starch and sucrose metabolism, amino sugar, and nucleotide sugar metabolism. The highest abundance was found in ABC transporters, and it belonged to membrane transport. In addition, both purine metabolism and pyrimidine metabolism belong to nucleotide metabolism. And 327 pathways were totally predicted in the cecum. As shown in Supplementary Figure 4a, level 2 of the KEGG pathway in the cecum was only replication and repair different in the ileum, and it was related to genetic information processing. The most abundant KEGG pathway in the cecum was the biosynthesis of amino acids (Supplementary Figure 4b). Ribosome (ko03010) and quorum sensing (ko02024) belonged to translation and cellular community-prokaryotes, respectively. Finally, there was no significant difference in the 2 and 3 levels of the top 10 KEGG pathways in the ileum and cecum (*p* > 0.05).

## Discussion

In terms of pig nutrition, an excessive supply of dietary calcium exceeding the actual requirement of pigs is not conducive to damage to the gastric barrier function of piglets (35). In this study, supplementation with different calcium or changing acidifier concentrations, neither alone nor in combination appeared to ultimately affect ADFI, ADG, or F:G in weaned piglets. Interestingly, multiple calcium in diet tended to reduce ADFI in the third and fourth weeks. However, the addition of organic trace minerals on the basis of calcium citrate did not change the ADFI and ADG within 6 weeks. Organic acids in feed have been reported to be effective growth promoters in pigs throughout the production cycle; due to the type and dosage of organic acids used, the timing of supplementation, type of diet and buffering capacity, hygiene and welfare standards, health status, animal age, and other factors, the response is quite different (36).

The results showed that the growth response range of weaned piglets was greater than that of aged animals. For example, in a meta-analysis study conducted by Tung and Pettigrew, the growth rate of piglets increased by 12.25 and 6.03%, respectively, in the first 2 or 4 weeks after weaning, while the growth rates of growing pigs (3.51%) or finishing pigs (2.69%) increased less (37). The study confirmed that piglets ingested a control diet without additional organic acids (30 g/kg of citric acid and 15 g/kg of fumaric acid) significantly higher than an acidified diet when a dietary choice was allowed (38). One study reported that citric acid supplementation did not affect ADG in a 4-week trial in weaned piglets (39). Another study reported no beneficial effect of incorporating citric acid on growth (40). The negative effect of citric acid on growth performance may be due to the reduced palatability of an acidifying diet.

Blood biochemical indicators can reflect the host's nutrient metabolism and pathological processes (41, 42). The contents of MDA, SOD, and GSH-Px in the blood reflect the level of the antioxidant defense system in animals (43). In this study, various sources of calcium and the addition of an acidifier did not significantly improve the antioxidant status of weaned piglets and did not change the immune indicators in the blood, so it did not regulate the immune system of piglets at the same time. At the same time, this study showed that adding organic trace elements on the basis of calcium citrate could not change the antioxidant and immune performance of weaned piglets. Dietary hydrated aluminosilicates have been shown to increase host serum ALT and ALP activities, which are associated with positive growth performance and muscle within reference ranges in pigs (44). Furthermore, serum ALP reflects skeletal development (45). Administration of organic acids has been reported to promote the production and activation of digestive enzymes (46). ALP is a brush border protein capable of hydrolyzing monophosphate. ALP is a marker of enterocyte differentiation, and a study has demonstrated that ALP can dephosphorylate and detoxify the endotoxin component of lipopolysaccharide, thereby maintaining intestinal homeostasis and inhibiting intestinal inflammation (47). Notably, in our study, the diet of calcium citrate instead of calcium carbonate markedly decreased ALT concentration, suggesting that dietary calcium carbonate content has a positive effect on piglets. In this experiment, when the piglets were fed the diet with calcium citrate as a calcium source, the content of ALP was significantly reduced after the acidifier was halved. Therefore, this showed that acidifiers actually play an important role in the growth and development of weaned piglets.

The intestinal microbiota plays a key role in the healthy growth and development of pigs, but the gut microbes are susceptible to many factors such as environment, stress, disease, and nutrition, leading to changes or imbalances in the gut microbiota. Diet is one of the main factors contributing to gut microbial colonization (48). This was evident from a study showing some significant differences in the gut microbial community structure of pigs after 2 weeks of feeding different experimental diets (49). Similarly, bacterial communities in feces were gradually rearranged both taxonomically and functionally after feeding four different diets that varied in protein source, calcium, and phosphorus concentrations (50). This suggested the criticality of diet for the regulation of the microbiota. However, the largest and most dynamic changes in microbiome transition occur during weaning (51). Alpha diversity, which refers to species diversity within a community, includes Chao1, Simpson, and Shannon (52). No significant differences were observed between species richness and α-diversity index of piglet ileal microbiota assigned to different calcium source groups in this study. Intestinal microbiota plays an important role in the maturation of the immune system and the efficient absorption/utilization of nutrients (53, 54). Lamendella et al. showed that Firmicutes and Bacteroidetes in the intestinal microorganisms of pigs are related to carbohydrate metabolism in the body (55). In this study, we found that at the phylum level, the microorganisms of Proteobacteria, Firmicutes, Verrucomicrobiota, and Campilobacterota accounted for more than 90% of the ileum microflora. Firmicutes, Bacteroidota, Actinobacteriota, and Proteobacteria were the main bacterial component of the cecum. A study reported that after 2 days post-weaning of piglets, intestinal *Lactobacillus* decreased sharply, while the number of coliforms increased (56). The *Lactobacillus* is an important probiotic, which may regulate intestinal flora, enhance immunity, improve intestinal function, and prevent diarrhea (57–59). In the experiment, we did not find the effect of different treatment groups on *Lactobacillus*. As shown in our study, *Firmicutes* are the dominant beneficial bacteria in the cecum, whereas *Proteobacteria* and *Actinobacillus* are usually considered pathogens. The results demonstrated that diets with different calcium sources did not regulate the abundance of *Firmicutes, Proteobacteria*, and *Actinobacillus*, thus not improving intestinal microecology. Decreasing acidifier from 1/1,000 to 0.5/1,000 without multiple calcium decreased Dorea, and after replacing calcium citrate with multiple calcium, Dorea was also significantly reduced, but this effect was not observed in the diet with half of the acidifier and multiple calcium. This suggested that the reduction of acidifier to the calcium citrate diet affected the intestinal microbial composition of the weaned piglets. Similarly, multiple calcium could also change the microbes in the hindgut of piglets compared to calcium citrate. Besides, calcium citrate and multiple calcium instead of calcium carbonate reduced the number of Subdoligranulum. It is possible that there is an explanation for the variation that different dietary components have different microbiomes, as the composition of the gut microbiome is known to be related to diet type (60). According to our results, the addition of organic trace minerals on the basis of calcium citrate did not change the main microbial composition in the intestine. This may be caused by too few organic trace mineral additives in the feed. Using marker gene data and a reference genome database obtained from 16S rRNA sequencing in this study, PICRUSt was used to predict the functional composition of the metagenome. The prediction results of gut microbiota revealed that the ileal and cecal microbiota were mainly involved in carbohydrate metabolism, membrane transport, amino acid metabolism, energy metabolism, nucleotide metabolism, and metabolism of cofactors and vitamins. According to the results of intestinal microbial function prediction, we anticipated that different calcium would not widely alter the gut microbiome.

## Conclusion

In summary, this study indicated that dietary supplementation with different calcium for weaned piglets could affect the ADFI and ADG of piglets. In addition, from the results of blood parameters, we inferred that dietary different calcium could not affect the immune and antioxidant status of piglets. However, the activities of ALT and ALP in blood were fluctuated among several different treatment groups. Our research provided a more comprehensive understanding of the intestinal microbial response of weaned piglets to different calcium sources, including the composition and functional potential of the bacteria in the intestine.

## Data Availability Statement

The datasets presented in this study can be found in online repositories. The names of the repository/repositories and accession number(s) can be found at: NCBI [accession: PRJNA815951].

## Ethics Statement

All experimental procedures involving animals were approved by the Laboratory Animal Welfare and Animal Experimental Ethical Inspection Committee at the Guangxi University (Nanning, China).

## Author Contributions

HS designed the experiment and revised the manuscript. AY and KW conducted the experiment. AY, KW, XP, FL, YiW, YC, YuW, DQ, and JZ collected and analyzed the data. AY and KW wrote the manuscript. All authors contributed to the article and approved the submitted version.

## Funding

The authors wish to acknowledge the financial support received from the National Natural Science Foundation of China (No. 31760746), the Science and Technology Major Project of Guangxi (China) (No. AA17204057), and the Key Research and Development Plan of Guatngxi (China) (No. AB19245037).

## Conflict of Interest

JZ is employed by Nanning Zeweier Feed Co., Ltd. The remaining authors declare that the research was conducted in the absence of any commercial or financial relationships that could be construed as a potential conflict of interest.

## Publisher's Note

All claims expressed in this article are solely those of the authors and do not necessarily represent those of their affiliated organizations, or those of the publisher, the editors and the reviewers. Any product that may be evaluated in this article, or claim that may be made by its manufacturer, is not guaranteed or endorsed by the publisher.

## Supplementary Material

Supplementary Figure 1 Distribution of top 30 ileal bacteria at genus level in weaned piglets.

Supplementary Figure 2 Distribution of top 30 cecal bacteria at genus level in weaned piglets.

Supplementary Figure 3 Top 10 predicted metagenomic functions at **(a)** level 2 and **(b)** level 3 of the KEGG pathways in the ileum. The bars stand for the percentage of relative abundance of each predicted function.

Supplementary Figure 4 Top 10 predicted metagenomic functions at **(a)** level 2 and **(b)** level 3 of the KEGG pathways in the cecum. The bars stand for the percentage of relative abundance of each predicted function.

Supplementary Table 1 Ingredients and composition of the basal diet for A (control).

## Abbreviations

A: Control
B, A (control) plus 5/1: 000 calcium citrate replace calcium carbonate
C, B plus 1/1: 000 organic trace minerals
D: C minus half of the acidifier
E, C plus 5/1: 000 multiple calcium replace calcium carbonate
MDA: malondialdehyde
SOD: superoxide dismutase
GSH-Px: glutathione peroxidase
ALT: ala-nine aminotransferase
AST: aspartate aminotransferase
AMY: amylase
ALP: alkaline phosphatase
LDH: lactate dehydrogenase.
