# Supplementary figures and images for: Effects of Different Sources of Calcium in the Diet on Growth Performance, Blood Metabolic Parameters, and Intestinal Bacterial Community and Function of Weaned Piglets
Source: Front Nutr. 2022 Apr 29;9:885497. doi: 10.3389/fnut.2022.885497 (PMC9101144; doi:10.3389/fnut.2022.885497)

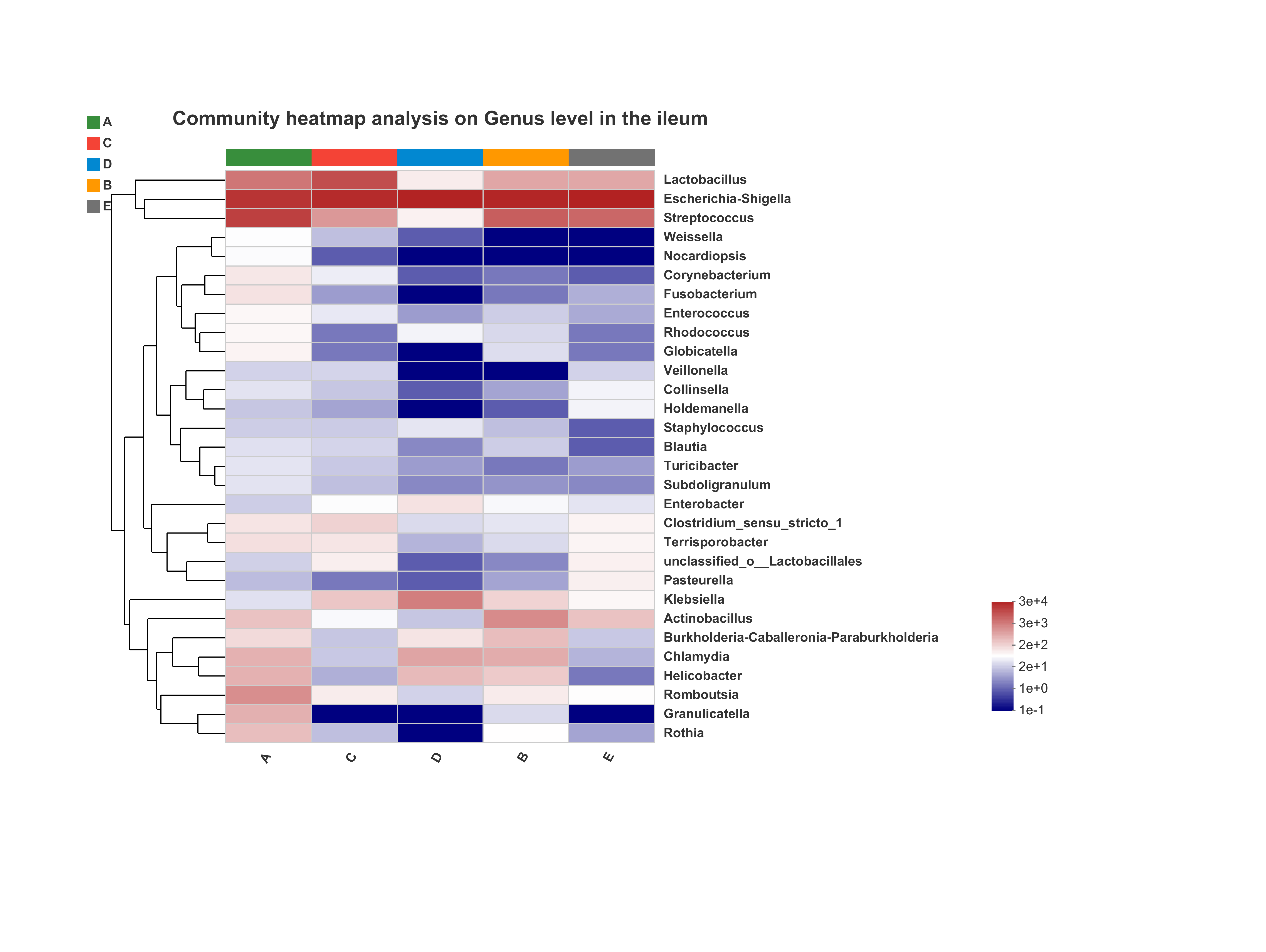

Supplement: Supplementary Figure 1 — Distribution of top 30 ileal bacteria at genus level in weaned piglets. [file Image_1.PNG]

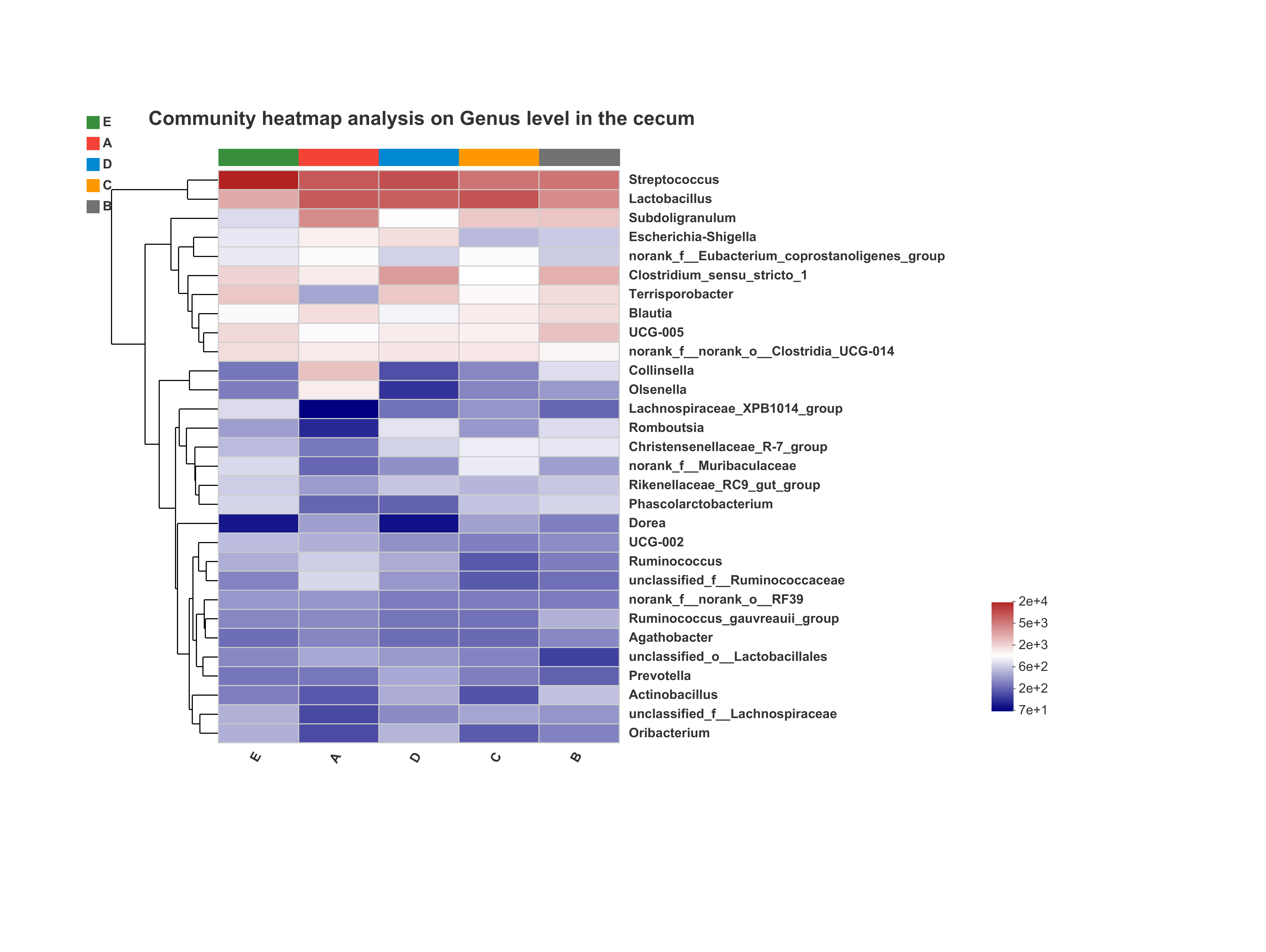

Supplement: Supplementary Figure 2 — Distribution of top 30 cecal bacteria at genus level in weaned piglets. [file Image_2.PNG]
