# Supplementary material for: Effects of Different Sources of Calcium in the Diet on Growth Performance, Blood Metabolic Parameters, and Intestinal Bacterial Community and Function of Weaned Piglets
Source: Front Nutr. 2022 Apr 29;9:885497. doi: 10.3389/fnut.2022.885497 (PMC9101144; doi:10.3389/fnut.2022.885497)
Supplement: Supplementary Table 1 — Ingredients and composition of the basal diet for A (control). [file Table_1.DOC]

**Table S1.** Ingredients and composition of the basal diet for A (control)

| Ingredients, % | 0-14d | 14-42d |
| --- | --- | --- |
| Corn | 61.43 | 65.02 |
| Soybean meal | 6.41 | 8.21 |
| Extruded soybean | 6.12 | 4.25 |
| Whey powder | 12.00 | 10.00 |
| Fish meal | 5.00 | 4.00 |
| SDPP | 5.00 | 3.00 |
| Limestone | 0.90 | 0.80 |
| Dicalcium phosphate | 0.40 | 0.54 |
| Salt | 0.35 | 0.30 |
| L-lysine HCl (98%) | 0.33 | 0.45 |
| DL-Methionine | 0.11 | 0.07 |
| L-Threonine | 0.11 | 0.13 |
| L-Tryptophan | 0.01 | 0.03 |
| Soy oil | 1.28 | 2.76 |
| ZnO | 0.20 | 0.04 |
| Vitamin and mineral premix^1^ | 0.35 | 0.40 |
| Nutrient composition (%) |  |  |
| DE (Kcal/kg) | 3455 | 3452 |
| CP | 19.41 | 19.06 |
| Calcium | 0.85 | 0.84 |
| Phosphorus | 0.32 | 0.32 |

Note: SDPP, Spray-dried plasma protein; Vitamin-mineral premix supplied per kg of feed: 100 mg of Fe (FeSO4), 100 mg of Zn (ZnSO4), 30 mg of Mn (MnSO4), 25 mg of Cu (CuSO4), 0.5 mg of I (KIO3), 0.3 mg of Co (CoSO4), 0.3 mg of Se (Na2SeO3), and 0.5 mg of ethoxyquin, 10,500 IU of vitamin A, 200 IU of vitamin D3, 60 IU of vitamin E, 2.0 mg of vitamin K3, 0.03 mg of vitamin B12, 12 mg of riboflavin, 30 mg of niacin, 25 mg of d-pantothenic acid, 0.18 mg of biotin, 1.5 mg of folic acid, 3.0 mg of thiamine, 2.25 mg of pyridoxine, 500 mg of choline chloride.
